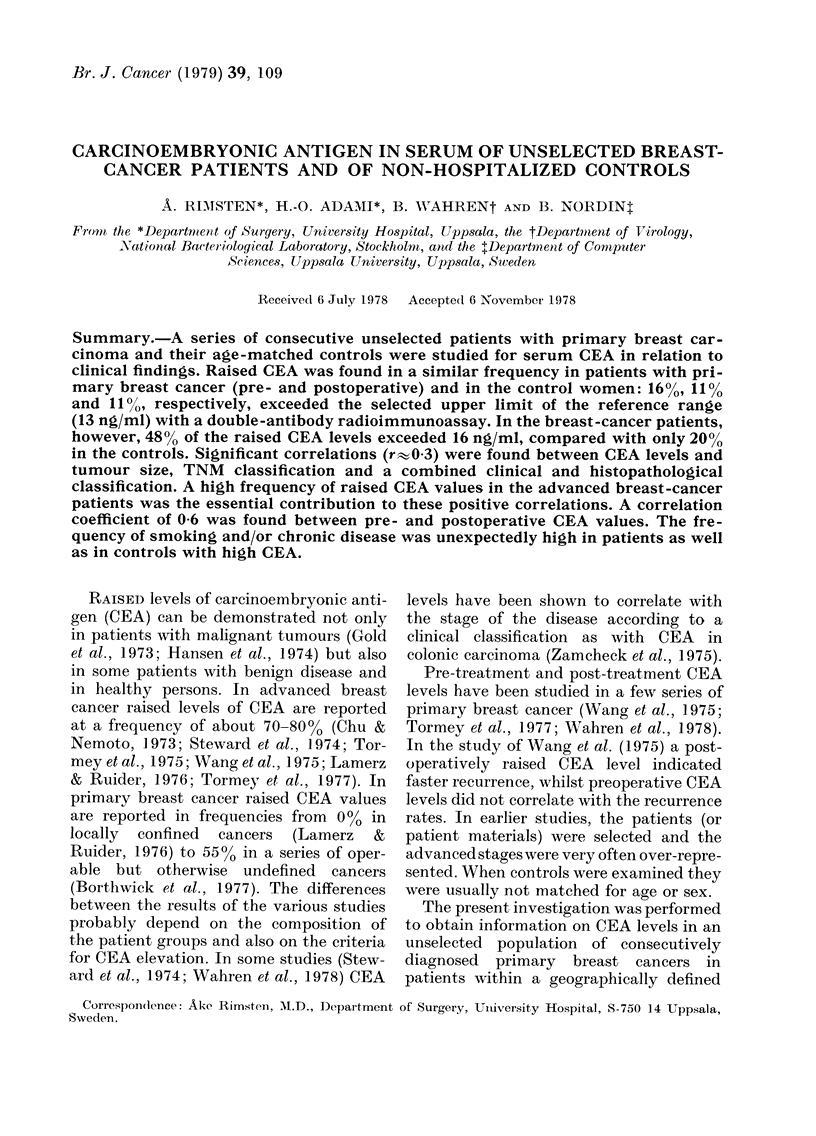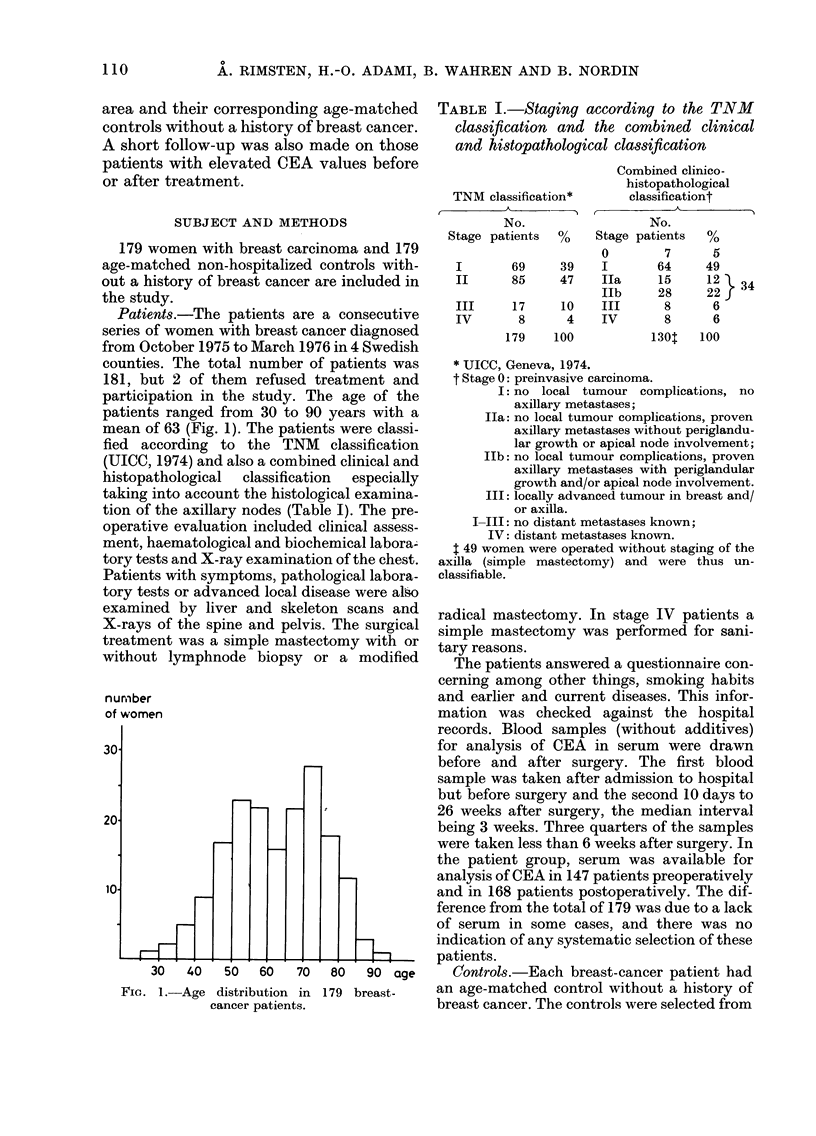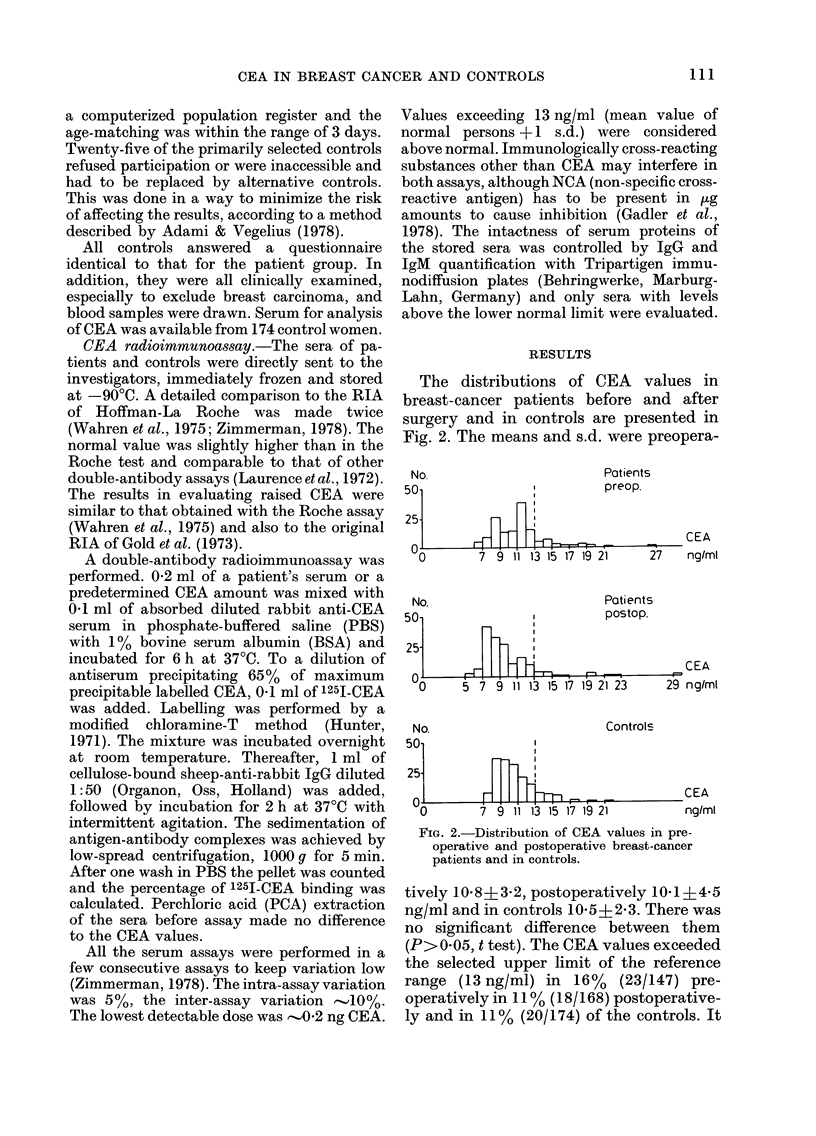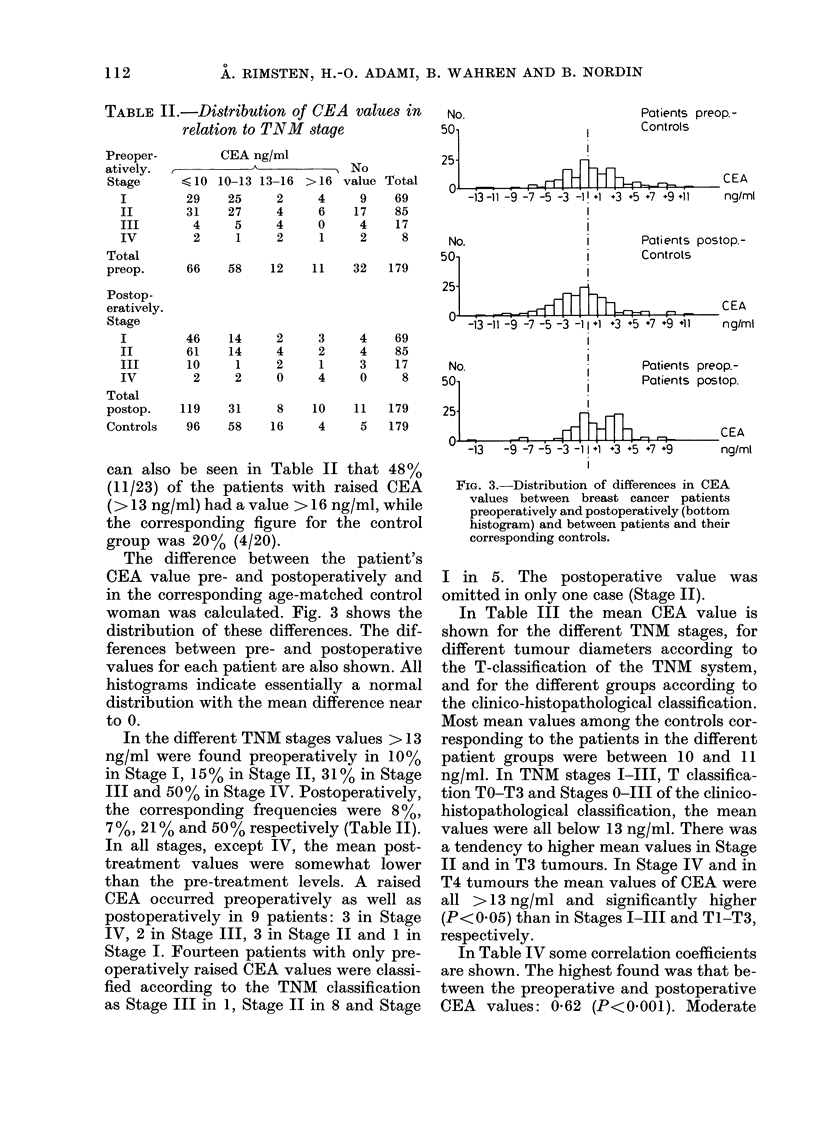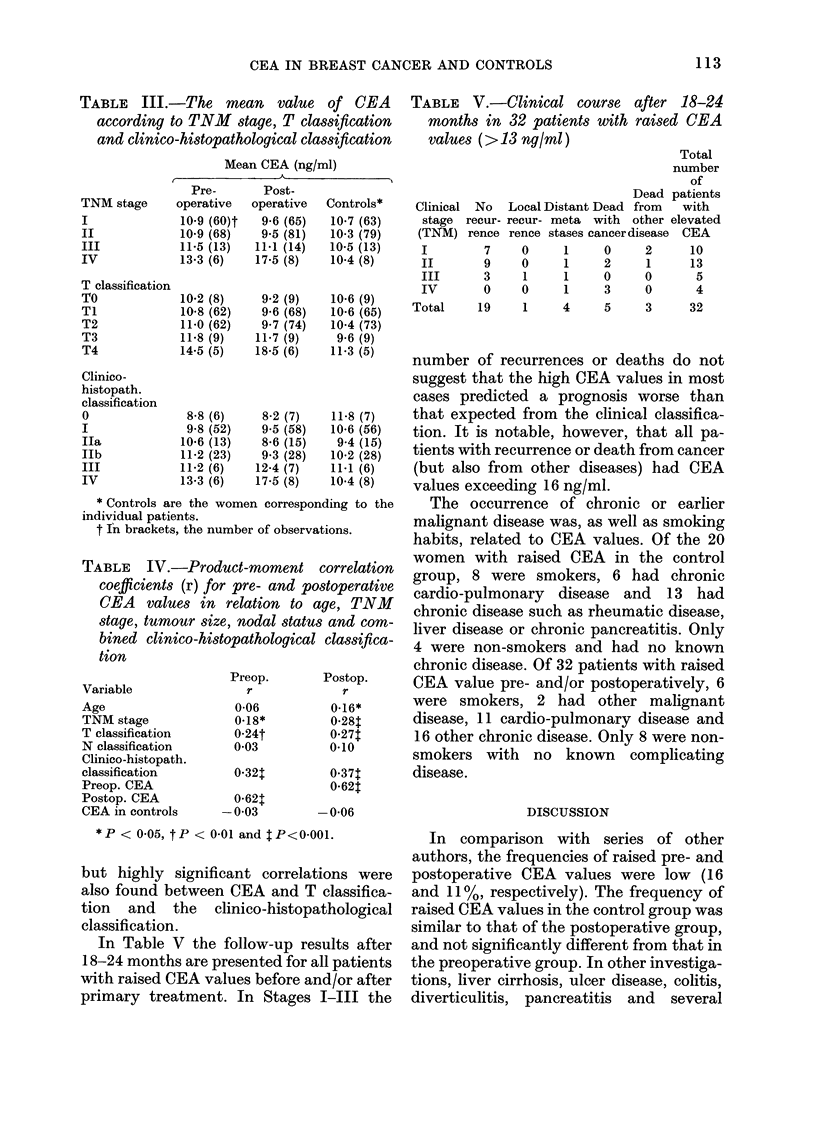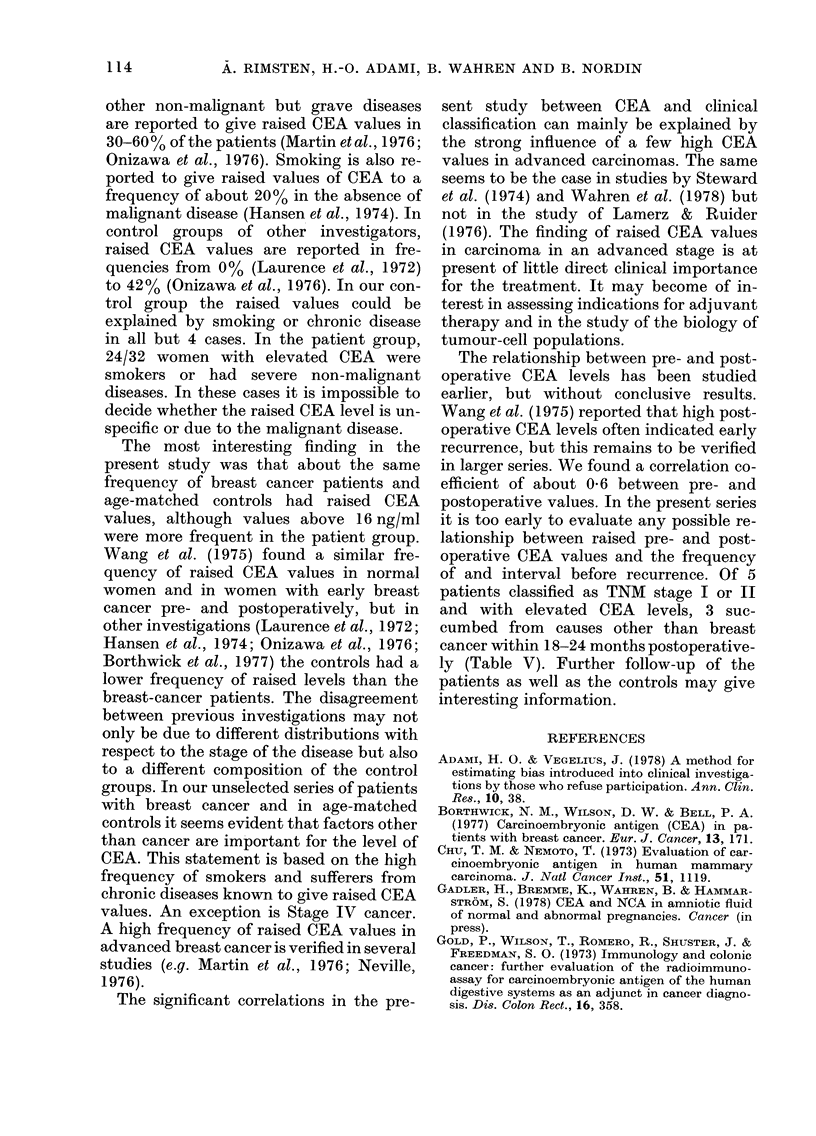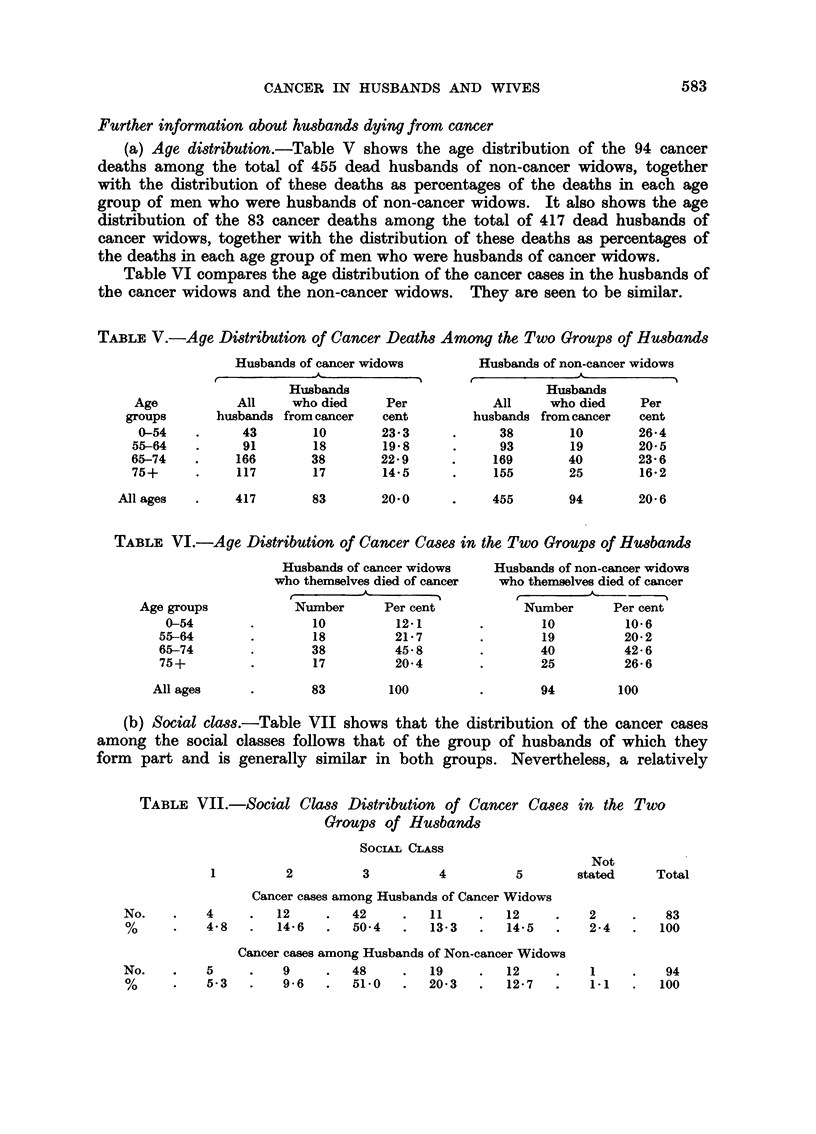# Carcinoembryonic antigen in serum of unselected breast-cancer patients and of non-hospitalized controls.

**DOI:** 10.1038/bjc.1979.20

**Published:** 1979-02

**Authors:** A. Rimsten, H. O. Adami, B. Wahren, B. Nordin

## Abstract

A series of consecutive unselected patients with primary breast carcinoma and their age-matched controls were studied for serum CEA in relation to clinical findings. Raised CEA was found in a similar frequency in patients with primary breast cancer (pre- and postoperative) and in the control women: 16%, 11% and 11%, respectively, exceeded the selected upper limit of the reference range (13 ng/ml) with a double-antibody radioimmunoassay. In the breast-cancer patients, however, 48% of the raised CEA levels exceeded 16 ng/ml, compared with only 20% in the controls. Significant correlations (r approximately 0.3) were found between CEA levels and tumour size, TNM classification and a combined clinical and histopathological classification. A high frequency of raised CEA values in the advanced breast-cancer patients was the essential contribution to these positive correlations. A correlation coefficient of 0.6 was found between pre- and postoperative CEA values. The frequency of smoking and/or chronic disease was unexpectedly high in patients as well as in controls with high CEA.


*Br. J. Cancer* (1979) **39**, 109

### CARCINOEMBRYONIC ANTIGEN IN SERUM OF UNSELECTED BREAST-CANCER PATIENTS AND OF NON-HOSPITALIZED CONTROLS

Å. RIMSTEN*, H.-O. ADAMI*, B. WAHREN† AND B. NORDIN‡

*From the *Department of Surgery, University Hospital, Uppsala, the †Department of Virology, National Bacteriological Laboratory, Stockholm, and the ‡Department of Computer Sciences, Uppsala University, Uppsala, Sweden*

Received 6 July 1978 Accepted 6 November 1978

**Summary.—A series of consecutive unselected patients with primary breast carcinoma and their age-matched controls were studied for serum CEA in relation to clinical findings. Raised CEA was found in a similar frequency in patients with primary breast cancer (pre- and postoperative) and in the control women: 16%, 11% and 11%, respectively, exceeded the selected upper limit of the reference range (13 ng/ml) with a double-antibody radioimmunoassay. In the breast-cancer patients, however, 48% of the raised CEA levels exceeded 16 ng/ml, compared with only 20% in the controls. Significant correlations ($r \approx 0{\cdot}3$) were found between CEA levels and tumour size, TNM classification and a combined clinical and histopathological classification. A high frequency of raised CEA values in the advanced breast-cancer patients was the essential contribution to these positive correlations. A correlation coefficient of 0·6 was found between pre- and postoperative CEA values. The frequency of smoking and/or chronic disease was unexpectedly high in patients as well as in controls with high CEA.**

RAISED levels of carcinoembryonic antigen (CEA) can be demonstrated not only in patients with malignant tumours (Gold *et al.*, 1973; Hansen *et al.*, 1974) but also in some patients with benign disease and in healthy persons. In advanced breast cancer raised levels of CEA are reported at a frequency of about 70–80% (Chu & Nemoto, 1973; Steward *et al.*, 1974; Tormey *et al.*, 1975; Wang *et al.*, 1975; Lamerz & Ruider, 1976; Tormey *et al.*, 1977). In primary breast cancer raised CEA values are reported in frequencies from 0% in locally confined cancers (Lamerz & Ruider, 1976) to 55% in a series of operable but otherwise undefined cancers (Borthwick *et al.*, 1977). The differences between the results of the various studies probably depend on the composition of the patient groups and also on the criteria for CEA elevation. In some studies (Steward *et al.*, 1974; Wahren *et al.*, 1978) CEA levels have been shown to correlate with the stage of the disease according to a clinical classification as with CEA in colonic carcinoma (Zamcheck *et al.*, 1975).

Pre-treatment and post-treatment CEA levels have been studied in a few series of primary breast cancer (Wang *et al.*, 1975; Tormey *et al.*, 1977; Wahren *et al.*, 1978). In the study of Wang *et al.* (1975) a postoperatively raised CEA level indicated faster recurrence, whilst preoperative CEA levels did not correlate with the recurrence rates. In earlier studies, the patients (or patient materials) were selected and the advanced stages were very often over-represented. When controls were examined they were usually not matched for age or sex.

The present investigation was performed to obtain information on CEA levels in an unselected population of consecutively diagnosed primary breast cancers in patients within a geographically defined

Correspondence: Åke Rimsten, M.D., Department of Surgery, University Hospital, S-750 14 Uppsala, Sweden.

area and their corresponding age-matched controls without a history of breast cancer. A short follow-up was also made on those patients with elevated CEA values before or after treatment.

### SUBJECT AND METHODS

179 women with breast carcinoma and 179 age-matched non-hospitalized controls without a history of breast cancer are included in the study.

*Patients.*—The patients are a consecutive series of women with breast cancer diagnosed from October 1975 to March 1976 in 4 Swedish counties. The total number of patients was 181, but 2 of them refused treatment and participation in the study. The age of the patients ranged from 30 to 90 years with a mean of 63 (Fig. 1). The patients were classified according to the TNM classification (UICC, 1974) and also a combined clinical and histopathological classification especially taking into account the histological examination of the axillary nodes (Table I). The preoperative evaluation included clinical assessment, haematological and biochemical laboratory tests and X-ray examination of the chest. Patients with symptoms, pathological laboratory tests or advanced local disease were also examined by liver and skeleton scans and X-rays of the spine and pelvis. The surgical treatment was a simple mastectomy with or without lymphnode biopsy or a modified

FIG. 1.—Age distribution in 179 breast-cancer patients.

TABLE I.—*Staging according to the TNM classification and the combined clinical and histopathological classification*

| TNM classification* | | | Combined clinico-histopathological classification† | | | |
|---|---|---|---|---|---|---|
| Stage | No. patients | % | Stage | No. patients | % | |
| | | | 0 | 7 | 5 | |
| I | 69 | 39 | I | 64 | 49 | |
| II | 85 | 47 | IIa | 15 | 12 | 34 |
| | | | IIb | 28 | 22 | |
| III | 17 | 10 | III | 8 | 6 | |
| IV | 8 | 4 | IV | 8 | 6 | |
| | 179 | 100 | | 130‡ | 100 | |

* UICC, Geneva, 1974.

† Stage 0: preinvasive carcinoma.
I: no local tumour complications, no axillary metastases;
IIa: no local tumour complications, proven axillary metastases without periglandular growth or apical node involvement;
IIb: no local tumour complications, proven axillary metastases with periglandular growth and/or apical node involvement.
III: locally advanced tumour in breast and/or axilla.
I–III: no distant metastases known;
IV: distant metastases known.

‡ 49 women were operated without staging of the axilla (simple mastectomy) and were thus unclassifiable.

radical mastectomy. In stage IV patients a simple mastectomy was performed for sanitary reasons.

The patients answered a questionnaire concerning among other things, smoking habits and earlier and current diseases. This information was checked against the hospital records. Blood samples (without additives) for analysis of CEA in serum were drawn before and after surgery. The first blood sample was taken after admission to hospital but before surgery and the second 10 days to 26 weeks after surgery, the median interval being 3 weeks. Three quarters of the samples were taken less than 6 weeks after surgery. In the patient group, serum was available for analysis of CEA in 147 patients preoperatively and in 168 patients postoperatively. The difference from the total of 179 was due to a lack of serum in some cases, and there was no indication of any systematic selection of these patients.

*Controls.*—Each breast-cancer patient had an age-matched control without a history of breast cancer. The controls were selected from a computerized population register and the age-matching was within the range of 3 days. Twenty-five of the primarily selected controls refused participation or were inaccessible and had to be replaced by alternative controls. This was done in a way to minimize the risk of affecting the results, according to a method described by Adami & Vegelius (1978).

All controls answered a questionnaire identical to that for the patient group. In addition, they were all clinically examined, especially to exclude breast carcinoma, and blood samples were drawn. Serum for analysis of CEA was available from 174 control women.

*CEA radioimmunoassay*.—The sera of patients and controls were directly sent to the investigators, immediately frozen and stored at −90°C. A detailed comparison to the RIA of Hoffman-La Roche was made twice (Wahren *et al.*, 1975; Zimmerman, 1978). The normal value was slightly higher than in the Roche test and comparable to that of other double-antibody assays (Laurence *et al.*, 1972). The results in evaluating raised CEA were similar to that obtained with the Roche assay (Wahren *et al.*, 1975) and also to the original RIA of Gold *et al.* (1973).

A double-antibody radioimmunoassay was performed. 0·2 ml of a patient's serum or a predetermined CEA amount was mixed with 0·1 ml of absorbed diluted rabbit anti-CEA serum in phosphate-buffered saline (PBS) with 1% bovine serum albumin (BSA) and incubated for 6 h at 37°C. To a dilution of antiserum precipitating 65% of maximum precipitable labelled CEA, 0·1 ml of $^{125}$I-CEA was added. Labelling was performed by a modified chloramine-T method (Hunter, 1971). The mixture was incubated overnight at room temperature. Thereafter, 1 ml of cellulose-bound sheep-anti-rabbit IgG diluted 1:50 (Organon, Oss, Holland) was added, followed by incubation for 2 h at 37°C with intermittent agitation. The sedimentation of antigen-antibody complexes was achieved by low-spread centrifugation, 1000 *g* for 5 min. After one wash in PBS the pellet was counted and the percentage of $^{125}$I-CEA binding was calculated. Perchloric acid (PCA) extraction of the sera before assay made no difference to the CEA values.

All the serum assays were performed in a few consecutive assays to keep variation low (Zimmerman, 1978). The intra-assay variation was 5%, the inter-assay variation ~10%. The lowest detectable dose was ~0·2 ng CEA. Values exceeding 13 ng/ml (mean value of normal persons +1 s.d.) were considered above normal. Immunologically cross-reacting substances other than CEA may interfere in both assays, although NCA (non-specific cross-reactive antigen) has to be present in μg amounts to cause inhibition (Gadler *et al.*, 1978). The intactness of serum proteins of the stored sera was controlled by IgG and IgM quantification with Tripartigen immunodiffusion plates (Behringwerke, Marburg-Lahn, Germany) and only sera with levels above the lower normal limit were evaluated.

### RESULTS

The distributions of CEA values in breast-cancer patients before and after surgery and in controls are presented in Fig. 2. The means and s.d. were preopera-

FIG. 2.—Distribution of CEA values in preoperative and postoperative breast-cancer patients and in controls.

tively 10·8±3·2, postoperatively 10·1±4·5 ng/ml and in controls 10·5±2·3. There was no significant difference between them ($P > 0{\cdot}05$, *t* test). The CEA values exceeded the selected upper limit of the reference range (13 ng/ml) in 16% (23/147) preoperatively in 11% (18/168) postoperatively and in 11% (20/174) of the controls. It

TABLE II.—*Distribution of CEA values in relation to TNM stage*

| Preoperatively. Stage | CEA ng/ml ⩽10 | 10–13 | 13–16 | >16 | No value | Total |
|---|---|---|---|---|---|---|
| I | 29 | 25 | 2 | 4 | 9 | 69 |
| II | 31 | 27 | 4 | 6 | 17 | 85 |
| III | 4 | 5 | 4 | 0 | 4 | 17 |
| IV | 2 | 1 | 2 | 1 | 2 | 8 |
| Total preop. | 66 | 58 | 12 | 11 | 32 | 179 |
| Postoperatively. Stage | | | | | | |
| I | 46 | 14 | 2 | 3 | 4 | 69 |
| II | 61 | 14 | 4 | 2 | 4 | 85 |
| III | 10 | 1 | 2 | 1 | 3 | 17 |
| IV | 2 | 2 | 0 | 4 | 0 | 8 |
| Total postop. | 119 | 31 | 8 | 10 | 11 | 179 |
| Controls | 96 | 58 | 16 | 4 | 5 | 179 |

can also be seen in Table II that 48% (11/23) of the patients with raised CEA (>13 ng/ml) had a value >16 ng/ml, while the corresponding figure for the control group was 20% (4/20).

The difference between the patient's CEA value pre- and postoperatively and in the corresponding age-matched control woman was calculated. Fig. 3 shows the distribution of these differences. The differences between pre- and postoperative values for each patient are also shown. All histograms indicate essentially a normal distribution with the mean difference near to 0.

In the different TNM stages values >13 ng/ml were found preoperatively in 10% in Stage I, 15% in Stage II, 31% in Stage III and 50% in Stage IV. Postoperatively, the corresponding frequencies were 8%, 7%, 21% and 50% respectively (Table II). In all stages, except IV, the mean post-treatment values were somewhat lower than the pre-treatment levels. A raised CEA occurred preoperatively as well as postoperatively in 9 patients: 3 in Stage IV, 2 in Stage III, 3 in Stage II and 1 in Stage I. Fourteen patients with only preoperatively raised CEA values were classified according to the TNM classification as Stage III in 1, Stage II in 8 and Stage I in 5. The postoperative value was omitted in only one case (Stage II).

FIG. 3.—Distribution of differences in CEA values between breast cancer patients preoperatively and postoperatively (bottom histogram) and between patients and their corresponding controls.

In Table III the mean CEA value is shown for the different TNM stages, for different tumour diameters according to the T-classification of the TNM system, and for the different groups according to the clinico-histopathological classification. Most mean values among the controls corresponding to the patients in the different patient groups were between 10 and 11 ng/ml. In TNM stages I–III, T classification T0–T3 and Stages 0–III of the clinico-histopathological classification, the mean values were all below 13 ng/ml. There was a tendency to higher mean values in Stage II and in T3 tumours. In Stage IV and in T4 tumours the mean values of CEA were all >13 ng/ml and significantly higher ($P<0{\cdot}05$) than in Stages I–III and T1–T3, respectively.

In Table IV some correlation coefficients are shown. The highest found was that between the preoperative and postoperative CEA values: 0·62 ($P<0{\cdot}001$). Moderate

TABLE III.—*The mean value of CEA according to TNM stage, T classification and clinico-histopathological classification*

| | Mean CEA (ng/ml) | | |
|---|---|---|---|
| TNM stage | Pre-operative | Post-operative | Controls* |
| I | 10·9 (60)† | 9·6 (65) | 10·7 (63) |
| II | 10·9 (68) | 9·5 (81) | 10·3 (79) |
| III | 11·5 (13) | 11·1 (14) | 10·5 (13) |
| IV | 13·3 (6) | 17·5 (8) | 10·4 (8) |
| T classification | | | |
| T0 | 10·2 (8) | 9·2 (9) | 10·6 (9) |
| T1 | 10·8 (62) | 9·6 (68) | 10·6 (65) |
| T2 | 11·0 (62) | 9·7 (74) | 10·4 (73) |
| T3 | 11·8 (9) | 11·7 (9) | 9·6 (9) |
| T4 | 14·5 (5) | 18·5 (6) | 11·3 (5) |
| Clinico-histopath. classification | | | |
| 0 | 8·8 (6) | 8·2 (7) | 11·8 (7) |
| I | 9·8 (52) | 9·5 (58) | 10·6 (56) |
| IIa | 10·6 (13) | 8·6 (15) | 9·4 (15) |
| IIb | 11·2 (23) | 9·3 (28) | 10·2 (28) |
| III | 11·2 (6) | 12·4 (7) | 11·1 (6) |
| IV | 13·3 (6) | 17·5 (8) | 10·4 (8) |

* Controls are the women corresponding to the individual patients.
† In brackets, the number of observations.

TABLE IV.—*Product-moment correlation coefficients* (r) *for pre- and postoperative CEA values in relation to age, TNM stage, tumour size, nodal status and combined clinico-histopathological classification*

| Variable | Preop. *r* | Postop. *r* |
|---|---|---|
| Age | 0·06 | 0·16* |
| TNM stage | 0·18* | 0·28‡ |
| T classification | 0·24† | 0·27‡ |
| N classification | 0·03 | 0·10 |
| Clinico-histopath. classification | 0·32‡ | 0·37‡ |
| Preop. CEA | | 0·62‡ |
| Postop. CEA | 0·62‡ | |
| CEA in controls | −0·03 | −0·06 |

* $P < 0·05$, † $P < 0·01$ and ‡ $P < 0·001$.

but highly significant correlations were also found between CEA and T classification and the clinico-histopathological classification.

In Table V the follow-up results after 18–24 months are presented for all patients with raised CEA values before and/or after primary treatment. In Stages I–III the number of recurrences or deaths do not suggest that the high CEA values in most cases predicted a prognosis worse than that expected from the clinical classification. It is notable, however, that all patients with recurrence or death from cancer (but also from other diseases) had CEA values exceeding 16 ng/ml.

TABLE V.—*Clinical course after 18–24 months in 32 patients with raised CEA values (>13 ng/ml)*

| Clinical stage (TNM) | No recurrence | Local recurrence | Distant metastases | Dead with cancer | Dead from other disease | Total number of patients with elevated CEA |
|---|---|---|---|---|---|---|
| I | 7 | 0 | 1 | 0 | 2 | 10 |
| II | 9 | 0 | 1 | 2 | 1 | 13 |
| III | 3 | 1 | 1 | 0 | 0 | 5 |
| IV | 0 | 0 | 1 | 3 | 0 | 4 |
| Total | 19 | 1 | 4 | 5 | 3 | 32 |

The occurrence of chronic or earlier malignant disease was, as well as smoking habits, related to CEA values. Of the 20 women with raised CEA in the control group, 8 were smokers, 6 had chronic cardio-pulmonary disease and 13 had chronic disease such as rheumatic disease, liver disease or chronic pancreatitis. Only 4 were non-smokers and had no known chronic disease. Of 32 patients with raised CEA value pre- and/or postoperatively, 6 were smokers, 2 had other malignant disease, 11 cardio-pulmonary disease and 16 other chronic disease. Only 8 were non-smokers with no known complicating disease.

### DISCUSSION

In comparison with series of other authors, the frequencies of raised pre- and postoperative CEA values were low (16 and 11%, respectively). The frequency of raised CEA values in the control group was similar to that of the postoperative group, and not significantly different from that in the preoperative group. In other investigations, liver cirrhosis, ulcer disease, colitis, diverticulitis, pancreatitis and several other non-malignant but grave diseases are reported to give raised CEA values in 30–60% of the patients (Martin *et al.*, 1976; Onizawa *et al.*, 1976). Smoking is also reported to give raised values of CEA to a frequency of about 20% in the absence of malignant disease (Hansen *et al.*, 1974). In control groups of other investigators, raised CEA values are reported in frequencies from 0% (Laurence *et al.*, 1972) to 42% (Onizawa *et al.*, 1976). In our control group the raised values could be explained by smoking or chronic disease in all but 4 cases. In the patient group, 24/32 women with elevated CEA were smokers or had severe non-malignant diseases. In these cases it is impossible to decide whether the raised CEA level is unspecific or due to the malignant disease.

The most interesting finding in the present study was that about the same frequency of breast cancer patients and age-matched controls had raised CEA values, although values above 16 ng/ml were more frequent in the patient group. Wang *et al.* (1975) found a similar frequency of raised CEA values in normal women and in women with early breast cancer pre- and postoperatively, but in other investigations (Laurence *et al.*, 1972; Hansen *et al.*, 1974; Onizawa *et al.*, 1976; Borthwick *et al.*, 1977) the controls had a lower frequency of raised levels than the breast-cancer patients. The disagreement between previous investigations may not only be due to different distributions with respect to the stage of the disease but also to a different composition of the control groups. In our unselected series of patients with breast cancer and in age-matched controls it seems evident that factors other than cancer are important for the level of CEA. This statement is based on the high frequency of smokers and sufferers from chronic diseases known to give raised CEA values. An exception is Stage IV cancer. A high frequency of raised CEA values in advanced breast cancer is verified in several studies (*e.g.* Martin *et al.*, 1976; Neville, 1976).

The significant correlations in the present study between CEA and clinical classification can mainly be explained by the strong influence of a few high CEA values in advanced carcinomas. The same seems to be the case in studies by Steward *et al.* (1974) and Wahren *et al.* (1978) but not in the study of Lamerz & Ruider (1976). The finding of raised CEA values in carcinoma in an advanced stage is at present of little direct clinical importance for the treatment. It may become of interest in assessing indications for adjuvant therapy and in the study of the biology of tumour-cell populations.

The relationship between pre- and postoperative CEA levels has been studied earlier, but without conclusive results. Wang *et al.* (1975) reported that high postoperative CEA levels often indicated early recurrence, but this remains to be verified in larger series. We found a correlation coefficient of about 0·6 between pre- and postoperative values. In the present series it is too early to evaluate any possible relationship between raised pre- and postoperative CEA values and the frequency of and interval before recurrence. Of 5 patients classified as TNM stage I or II and with elevated CEA levels, 3 succumbed from causes other than breast cancer within 18–24 months postoperatively (Table V). Further follow-up of the patients as well as the controls may give interesting information.

### REFERENCES

Adami, H. O. & Vegelius, J. (1978) A method for estimating bias introduced into clinical investigations by those who refuse participation. *Ann. Clin. Res.*, **10**, 38.

Borthwick, N. M., Wilson, D. W. & Bell, P. A. (1977) Carcinoembryonic antigen (CEA) in patients with breast cancer. *Eur. J. Cancer*, **13**, 171.

Chu, T. M. & Nemoto, T. (1973) Evaluation of carcinoembryonic antigen in human mammary carcinoma. *J. Natl Cancer Inst.*, **51**, 1119.

Gadler, H., Bremme, K., Wahren, B. & Hammarström, S. (1978) CEA and NCA in amniotic fluid of normal and abnormal pregnancies. *Cancer* (in press).

Gold, P., Wilson, T., Romero, R., Shuster, J. & Freedman, S. O. (1973) Immunology and colonic cancer: further evaluation of the radioimmunoassay for carcinoembryonic antigen of the human digestive systems as an adjunct in cancer diagnosis. *Dis. Colon Rect.*, **16**, 358.